# Identification of chronic mild traumatic brain injury using resting state functional MRI and machine learning techniques

**DOI:** 10.3389/fnins.2022.1099560

**Published:** 2023-01-09

**Authors:** Faezeh Vedaei, Najmeh Mashhadi, George Zabrecky, Daniel Monti, Emily Navarreto, Chloe Hriso, Nancy Wintering, Andrew B. Newberg, Feroze B. Mohamed

**Affiliations:** ^1^Department of Radiology, Jefferson Integrated Magnetic Resonance Imaging Center, Thomas Jefferson University, Philadelphia, PA, United States; ^2^Department of Computer Science and Engineering, University of California Santa Cruz, Santa Cruz, CA, United States; ^3^Department of Integrative Medicine and Nutritional Sciences, Marcus Institute of Integrative Health, Thomas Jefferson University, Philadelphia, PA, United States

**Keywords:** mild traumatic brain injury, machine learning, support vector machine, functional magnetic resonance imaging, resting-state

## Abstract

Mild traumatic brain injury (mTBI) is a major public health concern that can result in a broad spectrum of short-term and long-term symptoms. Recently, machine learning (ML) algorithms have been used in neuroscience research for diagnostics and prognostic assessment of brain disorders. The present study aimed to develop an automatic classifier to distinguish patients suffering from chronic mTBI from healthy controls (HCs) utilizing multilevel metrics of resting-state functional magnetic resonance imaging (rs-fMRI). Sixty mTBI patients and forty HCs were enrolled and allocated to training and testing datasets with a ratio of 80:20. Several rs-fMRI metrics including fractional amplitude of low-frequency fluctuation (fALFF), regional homogeneity (ReHo), degree centrality (DC), voxel-mirrored homotopic connectivity (VMHC), functional connectivity strength (FCS), and seed-based FC were generated from two main analytical categories: local measures and network measures. Statistical two-sample *t*-test was employed comparing between mTBI and HCs groups. Then, for each rs-fMRI metric the features were selected extracting the mean values from the clusters showing significant differences. Finally, the support vector machine (SVM) models based on separate and multilevel metrics were built and the performance of the classifiers were assessed using five-fold cross-validation and *via* the area under the receiver operating characteristic curve (AUC). Feature importance was estimated using Shapley additive explanation (SHAP) values. Among local measures, the range of AUC was 86.67–100% and the optimal SVM model was obtained based on combined multilevel rs-fMRI metrics and DC as a separate model with AUC of 100%. Among network measures, the range of AUC was 80.42–93.33% and the optimal SVM model was obtained based on the combined multilevel seed-based FC metrics. The SHAP analysis revealed the DC value in the left postcentral and seed-based FC value between the motor ventral network and right superior temporal as the most important local and network features with the greatest contribution to the classification models. Our findings demonstrated that different rs-fMRI metrics can provide complementary information for classifying patients suffering from chronic mTBI. Moreover, we showed that ML approach is a promising tool for detecting patients with mTBI and might serve as potential imaging biomarker to identify patients at individual level.

**Clinical trial registration:**

[clinicaltrials.gov], identifier [NCT03241732].

## Introduction

Traumatic brain injury (TBI) is one of the most common neurological disorders across the world that can result in a broad spectrum of symptoms that tremendously impact a person’s personality, behavior, thinking, and memory. In the United States it accounts for more than 2 million death and hospitalization and over 10 million worldwide (Taylor et al., 2017; Vedaei et al., 2021). TBI may happen as the result of multiple incidents including falls, vehicle accidents, athletic collisions, blast-related trauma, and abuse or assault. The majority of TBI cased are closed-head injuries, but some cases are open-head injuries, which occur when the skull is fractures or penetrated (Asken et al., 2018; O’Neill et al., 2018). Mild TBI (mTBI) is characterized by short-term and long-term clinical deficits including emotional and cognitive impairments in patients with subtle injury which only result in dysfunction of brain functional connectivity (FC) (O’Neill et al., 2018).

Resting-state functional magnetic resonance imaging (rs-fMRI) has been widely used in neuroscience for detecting intrinsic brain functional architecture as well as interactions between and within neural networks as the biomarkers of cognitive and neurological disorders (Jeter et al., 2013; Sours et al., 2015). Various approaches have been proposed for analyzing rs-fMRI data in mTBI cohorts including independent component analysis (ICA) (Bittencourt-Villalpando et al., 2021), graph theory (van der Horn et al., 2017), seed-based FC (Madhavan et al., 2019; Lemme et al., 2021; Philippi et al., 2021), amplitude of low-frequency fluctuation (ALFF), regional homogeneity (ReHo) (Zhan et al., 2015; Vedaei et al., 2021), degree centrality (DC) (Li et al., 2019), voxel-mirrored homotopic connectivity (VMHC) (Puig et al., 2020; Song et al., 2022). The growing body of studies of functional neuroimaging of the resting brain has shown that mTBI is accompanied by alterations of resting-state functional connectivity between and within intrinsic brain networks including the default mode network (DMN), fronto-parietal, motor, dorsal attention, and visual networks (Shumskaya et al., 2012; Stevens et al., 2012; Zhou et al., 2012, 2014; Dall’Acqua et al., 2017; Palacios et al., 2017; Liu et al., 2018; Li et al., 2019; Madhavan et al., 2019; Meier et al., 2020; Song et al., 2022). However, most of the studies were performed at the group level analysis which makes it challenging to generalize the findings to the identification of mTBI findings in individuals. Moreover, the consensus is far from certain regarding the use of different imaging metrics suggesting the need for a comprehensive study of various rs-fMRI metrics for depicting brain function alterations in mTBI patients. It has been proposed that various metrics may be complementary to each other in showing brain function alterations from different perspectives, thus providing more valuable information (Lv et al., 2018; Pang et al., 2021).

In recent years, machine-learning (ML) approaches as a branch of artificial intelligence have been used in clinical applications to facilitate predictive diagnoses and thereby help treatment plans (Ahmed et al., 2020). In neuroscience, ML algorithms have shown great promise in combining multimodal neuroimaging data and analyzing brain structural and functional alteration at the individual level, suggesting their high translational potential clinically (Janssen et al., 2018; Senders et al., 2018; Singh et al., 2022). The support vector machine (SVM), one of the popular supervised ML algorithms, recently has been increasingly used in the classification of neurodegenerative diseases applying to a range of MRI modalities and promising superior classification performance. It has been employed in patients’ classification tasks in a wide variety of neurological and psychiatric disorders including Parkinson’s disease (Zhang et al., 2020; Pang et al., 2021), Alzheimer’s disease (Schouten et al., 2016), traumatic brain injury (Vergara et al., 2017), bipolar disorder (Li H. et al., 2020), Schizophrenia (Wang et al., 2018; Liu et al., 2020), Obsessive-Compulsive Disorder (Jia et al., 2020), Epilepsy (Zhou et al., 2020; Wang et al., 2021), multiple sclerosis (Buyukturkoglu et al., 2021), and major depressive disorder (Sacchet et al., 2015).

The SVM has a great potential for transforming high-dimensional neuroimaging data into clinically effective decision-making criteria. It works based on constructing a separating hyperplane that maximizes the margin between the classes. In some cases that the dataset is not linearly separable in the original input space, the samples are mapped into a higher dimensional space using a kernel function to makese the classification easier in the transformed space. The commonly adopted kernel function is called a Gaussian radial basis function (RBF) which is corresponding to a non-linear SVM (RBF-SVM). The RBF-SVM is suitable for countless rs-fMRI features and for small sample sizes which in turn avoids over-fitting during classification (Amari and Wu, 1999; Noble, 2006; Pereira et al., 2009; Wang, 2009; Gravesteijn et al., 2020; Pang et al., 2021).

In the present study, several rs-fMRI metrics were measured and categorized in two types of features including local and network measurements. Local measures were voxel-based brain maps of fALFF, ReHo, DC, VMHC, and FC strength (FCS) and network measures including seed-based FC of brain networks (Ding et al., 2017). RBF-SVM classifier was employed on both local and network features using single level and combined rs-fMRI metrics. To the best of knowledge, our study is the first in classification of mTBI patients from healthy controls (HCs) using SVM and multilevel rs-fMRI metrics. We aimed to develop a non-invasive, automatic classification method to distinguish mTBI patients from HCs that can be translated into clinical practice as the imaging biomarker to identify patients at chronic state of mTBI. We hypothesized that each separate model is able to provide informative diagnostic performance. However, the combination of the multivariate metrics of rs-fMRI would lead to highest accuracy of classification.

## Materials and methods

### Participants

Sixty patients including 23 males (age: 46 ± 14.3 years) and 37 females (age: 45 ± 15.2 years) suffering from mTBI with chronic symptoms and forty matched HCs comprising 21 males (age: 41 ± 9.4 years) and 19 females (age: 39 ± 10.6 years) enrolled in this study after providing a written informed consent, approved by the local Institutional Review Board. Participants were recruited from local neurology offices and from the local community by self-referral. Exclusion criteria included if the patients had a history of other neurological disorders, significant medical illness, a current substance-use disorder, or current Diagnostic and Statistical Manual of Mental Disorders, 5th Edition (DSM-V) Axis I psychiatric illness. This study was registered on clinicaltrials.gov with the following identifier: NCT03241732. mTBI was defined according to the Mayo Classification System for Traumatic Brain Injury Severity, in which an injury was classified as mild if it met the following criteria: loss of consciousness <30 min, amnesia for <24 h, and no abnormal MRI findings (Malec et al., 2007). Enrolled patients had to report a history of one or more prior TBIs (one or multiple) meeting these criteria for mild TBI and have no structural injury to the brain such as a contusion, dura penetration, hematoma, or brainstem injury. They had to meet ICD-10 criteria for chronic mTBI (i.e., post-concussion syndrome) based upon symptoms that were the result of TBI and could include dysfunctionality such as cognitive problems, emotional problems (e.g., depression or anxiety), headache, dizziness, irritability, hypersensitivity to auditory or visual stimuli, balance problems, insomnia, or other subjective complaints specifically associated with the TBI. Also, patients had to report the chronic symptoms lasting for at least 6 months from the most recent TBI. For the HCs group, individuals were excluded if they had a history of previous TBI, a history of other neurological disorders, significant systemic medical illness, a current substance-use disorder, and current Diagnostic and Statistical Manual of Mental Disorders, 5th Edition (DSM-V) Axis I psychiatric illness.

### Imaging protocol

For each individual MRI examination was performed using a 3T Siemens Biograph mMR Positron Emission Tomography-MR (mMR PET-MR) scanner with a 32-channel head coil. A structural T1-weighted was acquired to check the lack of any sign of radiological findings of brain injury and to use during segmentation and registration steps of data preprocessing. MRI parameters for the anatomical T1-weighted sequence were as follows: repetition time = 1,600 msec, echo time = 2.46 msec, field of view (FOV) = 250 mm × 250 mm, matrix = 512 × 512, voxel size = 0.49 × 0.49, 176 slices with slice thickness = 1 mm.

Next, a resting-state BOLD scan was administered using an echo planar imaging (EPI) sequence using the imaging parameters including: FOV = 240 mm × 240 mm; voxel size = 3 mm × 3 mm × 4 mm; TR = 2,000.0 msec; TE = 30 msec; slice thickness = 4 mm; number of slices = 34; number of volumes = 180; and acquisition time = 366 s. During rs-fMRI, the participants were asked to close their eyes, and rest quietly without thinking about anything.

### Data processing

For all the participants, the rs-fMRI data was preprocessed using Data Processing Assistant for Resting-State fMRI (DPARSF. V6.1_220101^1^) (Yan et al., 2016; Vedaei et al., 2022). The preprocessing included the following steps: the first 10 volumes were discarded to allow magnetization to reach steady state and account for T1 relaxation effects. Then, the slice timing correction and head motion correction using six rigid body motion parameters were performed. Next, for each individual T1-weighted structural data and the mean of the realigned EPI images were co-registered and normalized to the EPI template in Montreal Neurological Institute (MNI) space with a resampling voxel size of 3 × 3 × 3 mm. Further, the Friston 24-parameter model (the 24 parameters including 6 head motion parameters, 6 head motion parameters of the previous scan, and the 12 corresponding squared items) was employed to regress out the micro head motion effects from the realigned data (Friston et al., 1996). No participants excluded from the study due to excessive head motion (>2.0 mm translation and/or 2.0° rotation) (Fox et al., 2005; Power et al., 2014). Further, signal from white matter and cerebrospinal fluid were regressed out and filtered with a temporal band-pass of 0.01–0.08 Hz to reduce the effects of low-frequency drifts and high-frequency respiratory and cardiac noise. The head motion was measured using frame-wise displacement (FD) and was not significantly different among mTBI and HCs groups (two-sample *t*-test, *p*-value = 0.205).

All data processing steps were limited within gray matter. Statistical parametric mapping 12^2^ was used to segment the brain to the gray matter for each participant. Then, the generated probabilistic map was binarized using fslmaths tools (cutoff = 0.2) to make the gray matter mask.

### Feature extraction

Different features were generated conducting different methods of rs-fMRI data processing. The features were categorized into two types including local measurements and network measurements. The features were extracted as the voxel-wise brain maps using DPARSF. V6.1_220101 and are detailed below. The motivation for extracting these number of rs-fMRI measurements were, first, because these are among the most common methods to analyze rs-fMRI data in neurodegenerative diseases and second, to evaluate the performance of SVM classification models by employing any of the local and network features as separate models as well as combining the local measures and network measures as multilevel measure models (Ding et al., 2017).

### Local measures

#### Fractional amplitude of low frequency fluctuation

For each participant, spatial smoothing [Gaussian kernel of full-width half maximum (FWHM) = 6 mm] was performed. Then, with the FFT, the time courses of rs-fMRI signal were converted to frequency domain, and the square root of the power spectrum was measured and averaged across the 0.01–0.08 Hz domain. Then, voxel-wise fALFF was measured as the ratio of power in low-frequency band (0.01–0.08 Hz) to the power of the entire frequency range (0–0.25 Hz). While ALFF describes the local spontaneous brain activity across the whole brain, by estimating the amplitude of neural activity in the low-frequency range (0.01–0.08 Hz), fALFF is a normalized derivation of ALFF representing the ratio of low-frequency range amplitudes (0.01–0.08 Hz) relative to the entire frequency range (e.g., 0–0.25 if TR = 2 s) amplitudes. As such, fALFF has been recommended to be used instead of ALFF due to its robustness against non-specific signal components such as physiological noise (Zou et al., 2008). To ensure standardization, for each participant, the fALFF of each voxel was transformed to *z*-scores using Fisher’s *z*-transform, and zfALFF maps were obtained (Zhou et al., 2014).

#### Regional homogeneity

Regional homogeneity was measured after band-pass filtering (0.01–0.08 Hz). This is accomplished on a voxel-based basis by calculating Kendall’s coefficient of concordance (KCC) for a given time series that is assigned as the center voxel with those of its nearest 26 neighboring voxels (Eq. 1) (Zang et al., 2004).


(1) $$w=\frac{\Sigma \,\left(R\,i\right)-n\,{\left(\bar{R\,i}\right)}^{2}}{\frac{1}{12}\,K^{2}\,\left(n^{3}-n\right)}$$

In this formula *w* is the KCC (range from 0 to 1) among given voxels; *K* is the number of neighboring voxels (*K* = 26); *R^–^_*i*_* is the mean rank across nearest neighbors (26 voxels) at the *ith* time point; and *n* is the total number of time points. For standardization purpose, ReHo value at each voxel was transformed to the standardized Fisher’s *Z*-transformation to obtain the zReHo maps. Spatial smoothing with an isotropic Gaussian kernel of 6 mm FWHM was performed after ReHo calculation.

#### Degree centrality

Degree centrality is a graph theory-based measurement that considers each voxel of the brain as a node and estimates how many edges it has with other nodes. As such, it measures functional connection between each voxel and any other voxel is defined as an edge. By computing Pearson correlation coefficients between time courses of each pair of voxels, a correlation matrix was firstly obtained. To remove the weak correlations that might be induced by noise, a threshold of *r* > 0.25 was used to obtain the undirected adjacency matrix. Then, for each voxel, the degree centrality was calculated as the sum the connections between this voxel with other voxels. For standardization purpose, the weighted DC was transformed to *z*-scores using Fisher’s *z*-transform. Finally, the zDC map was smoothed with an isotropic 6 mm FWHM Gaussian kernel (Pang et al., 2021; Wang et al., 2021).

#### Voxel-mirrored homotopic connectivity

Voxel-mirrored homotopic connectivity measures the synchrony in spontaneous activity between geometrically corresponding interhemispheric regions between pairs of symmetric voxels. It can be quantified by calculating the Pearson correlation coefficient between each voxel’s time series and that of its symmetric inter-hemispheric counterpart. For standardization purpose, correlation values were then transformed to *z*-scores using Fisher’s *z*-transform to generate zVMHC maps (Zuo et al., 2010; Pang et al., 2021).

#### Functional connectivity strength

The voxel-wise FC was measured by estimating Pearson’s correlations between the time series of any pairs of brain voxels within the gray matter mask. Then, for a given voxel i, FC was measured using the equation as follows (Eq. 2): (2):


(2) $$F\,C\,\left(i\right)=\frac{1}{N_{v\,o\,x\,e\,l\,s}-1}\,\sum_{j\neq i}Z\,i\,j,r\,i\,j>r\,0$$

where *z*_*ij*_ was the Fisher’s *Z*-transformed version of correlation coefficient, *r*_*ij*_, between voxel *i* and voxel *j*, and *r*_0_ was a correlation threshold that was used to exclude weak correlations possibly arising from noises (*r_0_* = 0.2 in this study). *r*_*ij*_ was converted to *z*_*ij*_ using Fisher’s *Z*-transformation. *N*_*voxels*_ was also defined as total number of voxels within the gray matter mask (Dai et al., 2015; Vedaei et al., 2022).

### Network measures

A total of 12 seed-based FC maps were computed from 6-mm spherical region of interests (ROIs). The seeds are supposed to be the center of the main brain functional networks and obtained from previously published locations, as summarized in Table 1 (Fox et al., 2005; Madhavan et al., 2019). For each seed, mean time series were measured from the pre-processed rs-fMRI data. Pearson correlation of the mean time series of each seed with every other voxel in the brain was computed to generate the corresponding FC map. The correlation maps were then transformed to *z*-scores using Fisher’s *z*-transform to produce 12 functional network maps for each participant.

**TABLE 1:** Seed location of 12 functional networks used in seed-based connectivity analysis.

Network	Seed region	MNI coordinates (*x*, *y*, *z*)
DMN	PCC	(2, −54, 26)
DAS	IPS R	(24, −60, 50)
ECN (L)	Left DLPFC	(−42, 34, 20)
ECN (R)	Right DLPFC	(44, 36, 20)
Motor dorsal (L)	Left precentral gyrus hand knob	(−28, −26, 64)
Motor dorsal (R)	Right precentral gyrus hand knob	(34, −24, 60)
Motor ventral (L)	Left precentral gyrus ventral	(−56, −6, 24)
Motor ventral (R)	Right precentral gyrus ventral	(60, −2, 24)
Salience (L)	Left anterior insula	(−32, 26, −14)
Salience (R)	Right anterior insula	(38, 22, −10)
Visual primary	BA17	(8, −78, 8)
Visual secondary	BA18	(−22, −90, 2)

DMN, default-mode network; DAS, dorsal attention network; ECN, executive control network; DLPFC, dorso-lateral prefrontal cortex; IPS, intraparietal sulcus; PCC, posterior cingulate cortex; L, left; R, right.

### Statistical analysis

In order to evaluate the distribution of gender and age within and between groups of mTBI and HCs, a chi-square and two-sample *t*-test were employed, respectively. A *p*-value of ≤0.05 was considered statistically significant. Voxel-based two-sample *t*-test were employed for group comparisons (mTBI versus HCs) on rs-fMRI metric maps including local and network measurements using the statistical analysis module in the Data Processing and Analysis of Brain Imaging (DPABI- V6.1_220101 toolbox^3^) (Yan et al., 2016). Gaussian random field (GRF) theory was employed for correction of multiple comparisons (voxel significance *p* < 0.001, cluster significance *p* < 0.01) controlling for age, gender, and mean FD were considered as covariates (Wang et al., 2018; Zhang et al., 2020; Gao et al., 2022). The clusters showing significant group differences were selected as ROIs. The average of rs-fMRI metrics including local and network measures were extracted over the mask of ROIs. The mean values were further utilized to prepare data frames and used as input features of the SVM algorithm following classification analysis. The name of the brain regions were reported based on the Automated Anatomical Labeling (AAL) atlas (Tzourio-Mazoyer et al., 2002).

### Classification and evaluation

The kernel-based RBF-SVM implemented on Anaconda platform^4^ with the “scikit-learn” package^5^ (Abraham et al., 2011) running on Python 3 was used as the classifier to examine the possibility of the combination of the clusters with significant differences to differentiate mTBI patients from HCs. For training and test datasets generation, data was split in the ratio of 80:20. Prior to training the classifiers, each feature in the training dataset was scaled using MinMaxScaler which modifies the dataset in a standardized scale with mean of 0 and a unit variance of 1 (Khatri and Kwon, 2022). The hyperparameters of C and gamma (C range: 10^–4^, 10^–3^, 10^–2^, 10^–1^, 1, 10, 10^2^, 10^3^, 10^4^; gamma range: 1, 0.1, 0.01, 0.001, 0.0001) were optimized using grid research *via* stratified nested five-fold cross validation (CV) in the training dataset. The C parameter assumed to control the tradeoff between empirical classification error and generalization of the model, while the gamma parameter defined the extent of influence of a single training example implemented in the kernel function. The parameter “class_weight” was set as “balanced” to deal with the sample imbalance. After the nested CV step, the RBF-SVM model with the optimal set of values of the hyperparameters was trained using the whole training dataset. The performance of the model was evaluated and quantified on the test dataset *via* five-fold stratified CV (repeated 100 times) using the receiver operator characteristic (ROC) curve analysis. The corresponding area under the curve (AUC), balanced accuracy, sensitivity, specificity, positive predictive value (PPV), and negative predictive value (NPV) were obtained.

**FIGURE 1:**
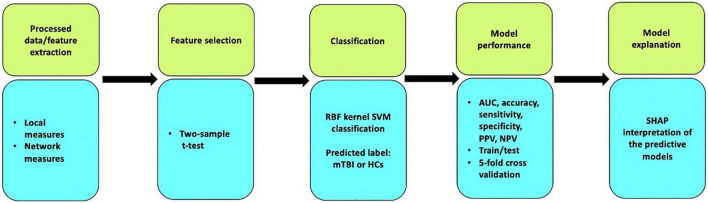
Workflow of the study including five main steps: data processing, feature extraction using statistical two-sample *t*-test, classification, model performance, and model interpretation.

During the multilevel measure analysis, the features were concatenated into a row vector (local and network measures, separately), the RBF-SVM was applied, and all the above-mentioned procedures executed to evaluate the performance of the classifier following multivariate analysis (Pettersson-Yeo et al., 2014; Lei et al., 2020). To interpret and understand the SVM model prediction, the Shapley Additive exPlanations (SHAP) algorithm was employed, which is a game-theoretic approach to measure how much each feature contributes to the model prediction by assigning an importance value to each feature corresponding to the probability of the diagnosis of chronic mTBI in this study (Lundberg and Lee, 2017). Further, 20 top features among local measures and network measures with the greatest contribution to the classification were reported. Figure 1 shows the workflow of the study.

## Results

### Demographic characteristics

Demographic statistical analysis showed a significant difference between the age of HCs and mTBI groups (two-sample *t*-test, *p*-value = 0.03). However, no significant difference in the proportion of males and females was found in each group (HCs: chi-square, χ^2^ = 0.1, *p*-value = 0.75; mTBI: chi-square, χ^2^ = 3.6, *p*-value = 0.08) (Table 2).

**TABLE 2:** Demographic of participants in the mTBI and HCs groups.

	HCs	mTBI	*P*-value	Statistic
Demographics	*n* = 40	*n* = 60		
Age (year) (SD)	40.3 (9.9)	46.0 (14.8)	0.03^a^	*T* = −2.1^a^
Sex (M/F)	21: 19	23: 37		
			CN: 0.75^b^	χ^2^ (CN): 0.1^b^
			mTBI: 0.08^b^	χ^2^ (mTBI): 3.6^b^
Injury-to-imaging interval (95% lower CI–95% upper CI) (months)	–	24–37		
Single concussion vs. multiple (single: multiple)	–	17: 43		

HCs, healthy controls; mTBI, mild traumatic brain injury; SD, standard deviation; CI, confidence interval. ^a^*p*-value and *T*-statistic obtained by two-sample *t*-test. ^b^*p*-value and χ^2^-statistic obtained using chi-square *t*-test.

### Group differences in rs-fMRI measures

**FIGURE 2:**
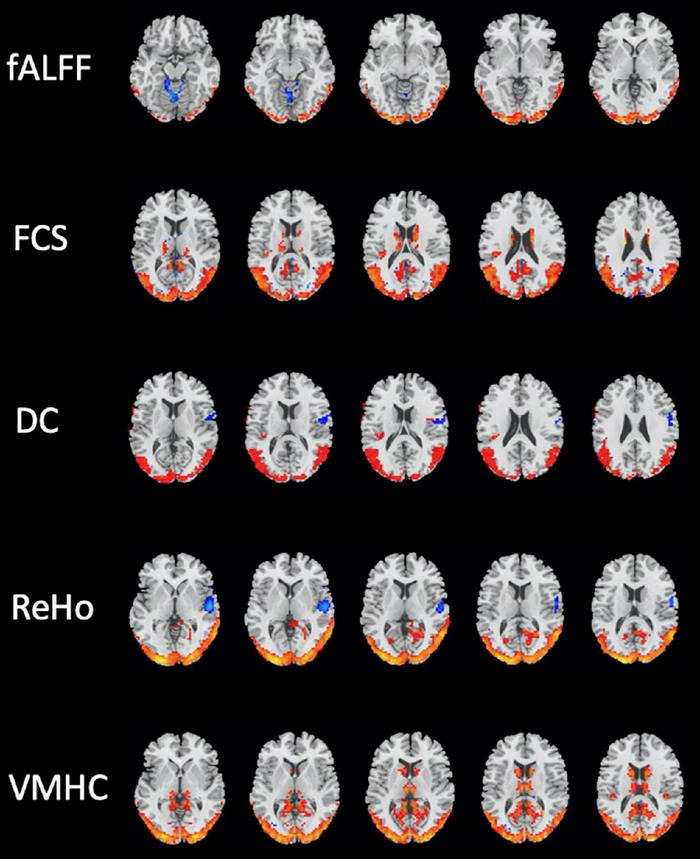
Brain regions with significant differences in local resting-state functional magnetic resonance imaging (rs-fMRI) measures between the mild traumatic brain injury (mTBI) and healthy controls (HCs) groups (two-sample *t*-test, GRF-corrected, voxel-level *p* < 0.001, cluster-level *p* < 0.01).

**FIGURE 3:**
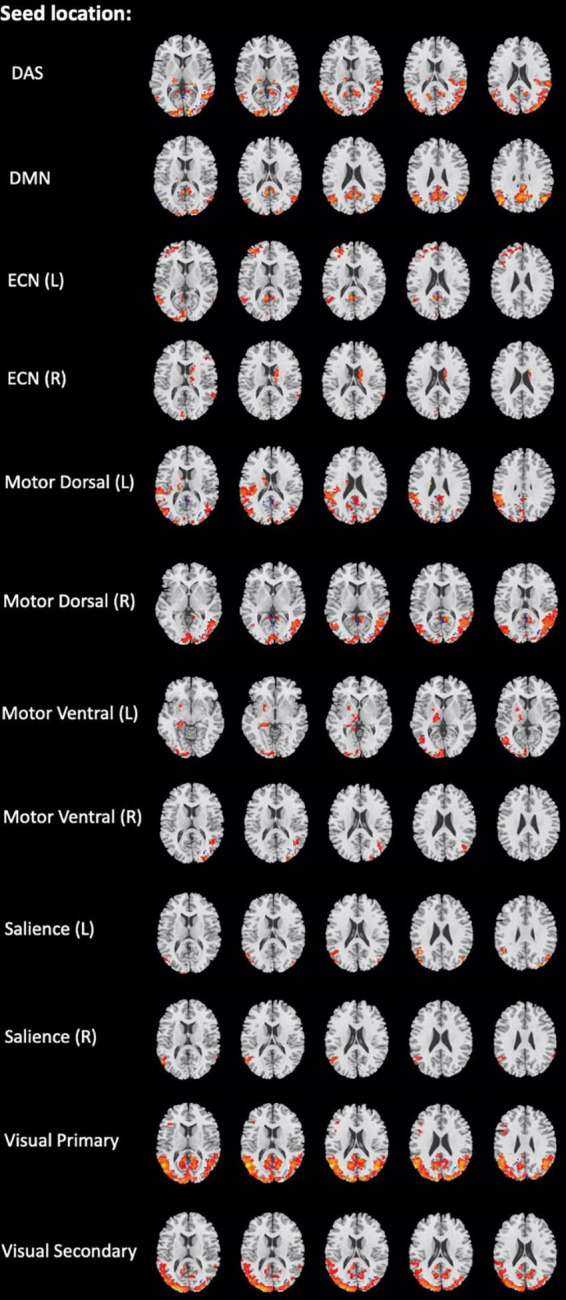
Brain regions with significant differences in the network seed-based functional connectivity measures between the mild traumatic brain injury (mTBI) and healthy controls (HCs) groups (two-sample *t*-test, GRF-corrected, voxel-level *p* < 0.001, cluster-level *p* < 0.01).

Several brain regions have been found as significant differences between the mTBI and HCs groups for each local and network measure. The detail of the results of two-sample *t*-tests comparing between the groups for the local and network measures are summarized in Tables 3, 4, and shown in Figures 2, 3, respectively.

**TABLE 3:** Brain regions with significant local measure differences between the mTBI patients and HCs.

Local measure	Brain region (AAL)	Voxels	Peak MNI coordinate (x, y, z)	*T*-value
fALFF	Occipital_Mid_L	2,539	(−36, −78, 36)	6.77
	Cerebellum_Crus1_L	434	(−33, −63, −36)	-3.18
	Cingulate_Mid_L	72	(0, −30, 33)	5.70
	Precuneus_R	62	(9, −57, 24)	4.10
	Cerebellum_8_R	60	(27, −45, −45)	-3.18
	Rectus_R	48	(6, 42, −24)	-3.18
	Calcarine_L	38	(−9, −54, 6)	4.48
	Precentral_R	32	(39, 0, 48)	-3.19
FCS	Occipital_Mid_L	5,983	(−18, −12, 27)	8.61
	Frontal_Mid_L	134	(−30, 24, 60)	4.32
	Frontal_Sup_R	111	(33, 0, 69)	4.37
DC	Occipital_Mid_R	2,706	(33, −99, 3)	33.27
	Cerebellum_4_5_L	368	(9, −36, −24)	4.14
	Fusiform_L	162	(−36, −30, −15)	26.00
	Rolandic_Oper_R	117	(36, −3, 18)	10.11
	Postcentral_L	96	(−51, 21, 48)	19.22
	Temporal_Sup_L	84	(−72, −24, 3)	11.78
	Fusiform_L	77	(−24, 9, −45)	12.22
	Rectus_L	55	(−9, 60, −24)	4.80
	Calcarine_R	54	(27, −66, 12)	-3.17
ReHo	Occipital_Mid_L	4,753	(−33, −87, 33)	7.08
	Frontal_Sup_L	2,018	(−15, −9, 72)	-3.17
	Fusiform_R	726	(30, −54, −3)	5.61
	Temporal_Sup_R	603	(48, −9, −15)	-3.17
	Frontal_Sup_Medial_L	207	(3, 57, 33)	-3.17
	Lingual_L	186	(−24, −69, −12)	5.11
	OFCpost_L	178	(−27, 21, −27)	−3.17
	Putamen_L	154	(−24, 0, 6)	5.55
VMHC	Occipital_Mid_L	3,058	(−27, −93, 0)	7.76
	Calcarine_R	2,968	(27, −93, 0)	7.76
	Cerebellum_Crus2_R	180	(18, −84, −42)	5.84
	Cerebellum_9_L	68	(−6, −45, −48)	4.85
	Rolandic_Oper_R	57	(39, −30, 18)	4.53
	Rolandic_Oper_L	56	(−39, −30, 18)	4.53
	Supp_Motor_Area_R	51	(9, 21, 48)	4.95
	Cerebellum_9_R	48	(6, −45, −48)	4.85
	Parietal_Sup_R	48	(39, −15, 72)	4.02
	Frontal_Sup_L	43	(−9, 21, 48)	4.95

fALFF, fractional amplitude of low-frequency fluctuations; FCS, functional connectivity strength; DC, degree centrality; ReHo, regional homogeneity; VMHC, voxel-mirrored homotopic connectivity; mTBI, mild traumatic brain injury; HCs, healthy controls; ALL, automated anatomical labeling; MNI, Montreal Neurological Institute; T, statistical value of peak voxel. *x, y, z*, coordinates of primary peak locations in the space of MNI; L, left; R, right.

**TABLE 4:** Brain regions with significant network measure differences between the mTBI patients and HCs.

Network-seed	Brain region (AAL)	Voxels	Peak MNI coordinate (*x*, *y*, *z*)	*T*-value
DAS	Temporal_Mid_R	1,354	(51, −60, 0)	5.49
	Occipital_Inf_L	895	(−18, −99, −9)	5.44
	Precuneus_R	190	(15, −30, 12)	5.56
	ParaHippocampal_R	103	(36, −18, −24)	4.98
	Parietal_Sup_R	80	(24, −51, 57)	4.08
DMN	Precuneus_R	1,973	(15, −54, 39)	5.73
	Angular_L	603	(−42, −72, 36)	6.01
	Calcarine_R	427	(15, −99, 3)	5.38
	Angular_R	370	(48, −66, 36)	5.73
	Temporal_Mid_L	77	(−63, −18, −15)	4.50
	Frontal_Sup_Medial_L	76	(−6, 33, 63)	3.96
	Cerebellum_9_R	75	(3, −54, −48)	5.27
ECN (L)	Temporal_Mid_L	431	(−66, −39, −12)	5.99
	Frontal_Mid_L	361	(−27, 45, 12)	5.61
	Occipital_Mid_L	226	(−27, −99, 6)	5.21
	Frontal_Mid_L	206	(−45, 18, 39)	5.13
	Temporal_Inf_R	200	(60, −57, −9)	5.88
	Cerebellum_Crus1_R	126	(36, −63, −33)	5.75
	Precuneus_L	89	(−6, −51, 18)	4.93
ECN (R)	Temporal_Inf_R	372	(60, −54, −15)	5.95
	Calcarine_L	200	(−6, −93, 9)	5.17
	Caudate_R	139	(18, −6, 27)	7.78
	Cerebellum_8_R	127	(27, −66, −51)	4.75
	Frontal_Inf_Tri_R	84	(45, 36, 6)	4.79
Motor dorsal (L)	Angular_L	2,055	(−48, −51, 33)	5.84
	Hippocampus_L	182	(−18, −12, 27)	6.83
	Precuneus_R	122	(9, −48, 9)	4.17
	Temporal_Inf_L	84	(−42, −33, −24)	4.28
	Precentral_L	76	(−21, −12, 78)	3.95
Motor dorsal (R)	Cuneus_R	2,423	(24, −84, 45)	5.44
	Precuneus_R	205	(6, −51, 15)	5.10
Motor ventral (L)	Angular_L	429	(−45, −54, 30)	4.55
	Occipital_Mid_L	190	(−27, −96, 0)	4.64
	Putamen_L	154	(−18, −15, 27)	6.25
	Temporal_Inf_L	134	(−45, −42, −24)	5.15
	Rolandic_Oper_L	90	(−39, −18, 18)	5.02
	Cerebellum_8_L	83	(−12, −60, −51)	4.91
Motor ventral (R)	Occipital_Mid_R	189	(33, −93, 0)	4.55
	Temporal_Sup_L	99	(−57, −9, 6)	−3.18
	Temporal_Mid_R	84	(45, −78, 21)	4.13
Salience (L)	Temporal_Mid_L	152	(−45, −57, 24)	4.91
	Occipital_Mid_R	144	(30, −87, 33)	5.20
	Angular_L	77	(−48, −66, 45)	4.51
	Calcarine_L	74	(−9, −96, −9)	4.67
Salience (R)	Temporal_Sup_R	170	(54, −3, −15)	4.67
	Fusiform_R	157	(42, −30, −24)	5.08
	Temporal_Mid_L	132	(−54, −63, 15)	4.51
	Temporal_Mid_R	103	(63, −54, −33)	4.63
	Occipital_Mid_L	75	(−30, −87, 27)	4.56
Visual primary	Angular_L	4,195	(54, −66, 24)	6.71
	Frontal_Mid_L	375	(−45, 6, 54)	5.01
	Temporal_Inf_L	124	(−51, −45, −21)	5.82
	Fusiform_R	82	(45, −30, −24)	6.01
Visual secondary	Occipital_Inf_L	3,849	(−30, −90, −9)	7.55
	Postcentral_L	248	(−48, −30, 57)	4.57

DAS, dorsal attention; DMN, default mode network; ECN, executive control network; mTBI, mild traumatic brain injury; HCs, healthy controls; ALL, automated anatomical labeling; MNI, Montreal Neurological Institute; T, statistical value of peak voxel. *x, y, z*, coordinates of primary peak locations in the space of MNI; L, left; R, right.

### Classification performance

**FIGURE 4:**
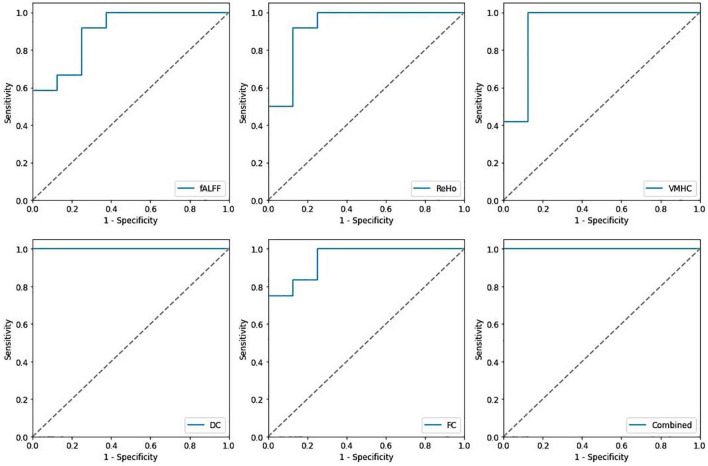
Receiving operating characteristic curve (ROC) of the support vector machine (SVM) model based on the single and combined local measures.

**FIGURE 5:**
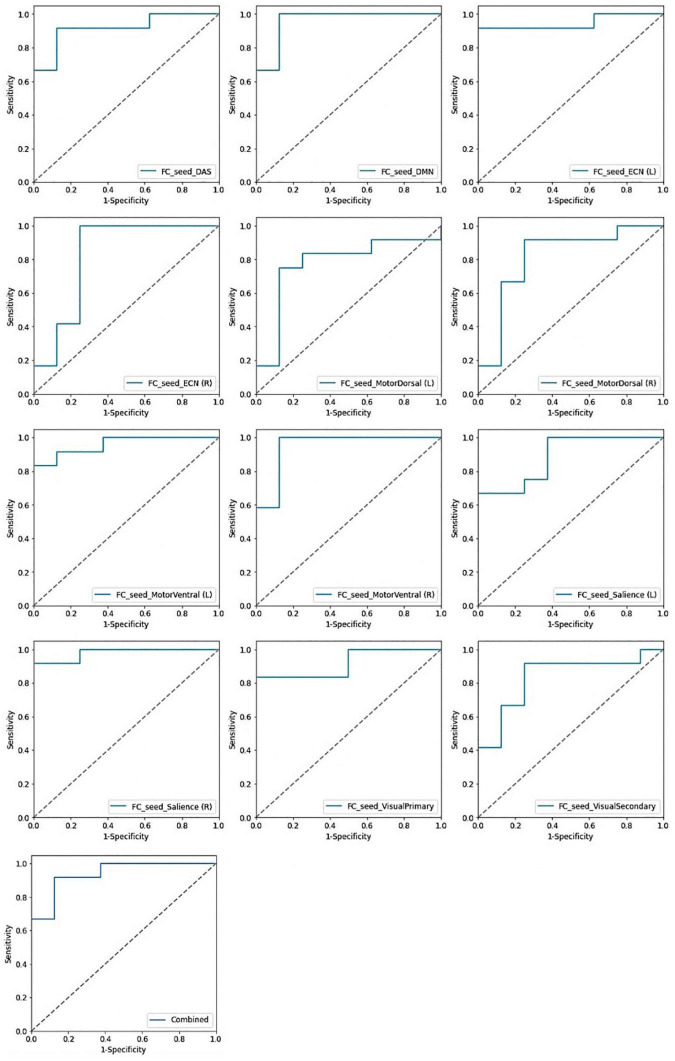
Receiving operating characteristic curve (ROC) of the support vector machine (SVM) model based on the single and combined network measures.

In identification of mTBI patients versus HCs, the %AUC, balanced accuracy, sensitivity, specificity, PPV, and NPV values were obtained for single and combined local and network measures. The list of these values and ROC analysis are summarized in Table 5 and Figures 4, 5. The %AUC value for local measures was 88.75 for fALFF, 96.25 for ReHo, 100 for DC, 96.25 for VMHC, and 86.67 for FCS. Also, the %AUC for the network measures was 86.25 for the default mode network (DMN), 82.5 for dorsal attention network (DAS), 80.42 for both left and right executive control network (ECN), 86.67 and 82.5 for left and right motor dorsal, respectively; 81.67 and 93.33 for left and right motor ventral, respectively; 82.92 and 81.25 for left and right salience network, respectively; 82.08 for visual primary; and 97.5 for visual secondary. Additionally, we found highest performance of SVM classification using multilevel measure analysis achieving an %AUC of 100 for both the local and network measures. However, DC as a single measure showed the highest %AUC of 100 among the whole rs-fMRI metrics.

**TABLE 5:** Classification performances of single metric model and combined model for local and network rs-fMRI metrics.

	Metrics	AUC (%)	B-ACC (%)	Sensitivity (%)	Specificity (%)	PPV (%)	NPV (%)
Local metrics	fALFF	88.75	88.75	95.00	82.50	89.19	92.14
	ReHo	96.25	96.25	100	92.50	95.60	100
	DC	100	100	100	100	100	100
	VMHC	96.25	96.25	100	92.50	95.60	100
	FCS	86.67	86.67	98.33	75.00	86.86	97.78
	Combined	100	100	100	100	100	100
Network metrics	DMN	86.25	86.25	100	72.50	84.75	100
	DAS	82.50	82.50	95.00	70.00	83.44	91.79
	ECN (L)	80.42	80.42	98.33	62.50	81.57	97.50
	ECN (R)	80.42	80.42	98.33	62.50	82.01	97.50
	Motor dorsal (L)	86.67	86.67	93.33	80.00	89.29	92.32
	Motor dorsal (R)	82.50	82.50	90.00	75.00	85.71	85.84
	Motor ventral (L)	81.67	81.67	93.33	70.00	82.89	88.45
	Motor ventral (R)	93.33	93.33	96.67	90.00	93.85	94.92
	Salience (L)	82.92	82.92	93.33	72.50	83.83	87.98
	Salience (R)	81.25	81.25	90.00	72.50	84.37	86.70
	Visual primary	83.33	83.33	91.67	75.00	86.40	90.00
	Visual secondary	82.08	82.08	81.67	82.50	88.98	74.71
	Combined	97.50	97.50	100	95.00	96.92	100

AUC, area under the receiver operator curve; B-ACC, balanced accuracy; PPV, positive predictive value; NPV, negative predictive value; fALFF, fractional amplitude of low frequency fluctuation; ReHo, regional homogeneity; DC, degree centrality; VMHC, voxel mirrored homotopic connectivity; FCS, functional connectivity strength; DMN, default mode network; DAS, dorsal attention network; ECN, executive control network; L, left; R, right.

### Brain regions with the greatest contribution to classification

**FIGURE 6:**
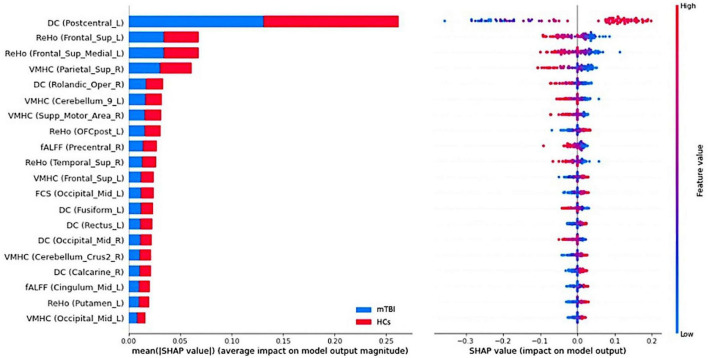
Summary plot of the top 20 features with the most contribution to the model prediction of the local measures. **(Left)** The mean absolute Shapley Additive explanations (SHAP) values of the features. **(Right)** The SHAP values of features in every sample. Each line represents a feature, and each dot visualizes the SHAP value for one subject and corresponding feature. A positive SHAP value indicates an increase in the risk of predicting mild traumatic brain injury (mTBI) and vice versa. The vertical axis represents both the features, ordered by the mean absolute SHAP values and their distribution.

**FIGURE 7:**
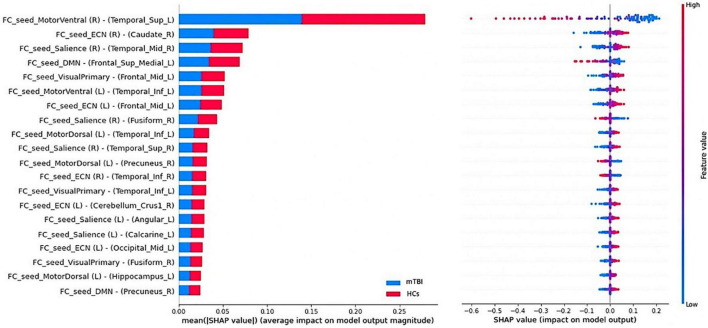
Summary plot of the top 20 features with the most contribution to the model prediction of the network measures. **(Left)** The mean absolute Shapley Additive explanations (SHAP) values of the features. **(Right)** The SHAP values of features in every sample. Each line represents a feature, and each dot visualizes the SHAP value for one subject and corresponding feature. A positive SHAP value indicates an increase in the risk of predicting mild traumatic brain injury (mTBI) and vice versa. The vertical axis represents both the features, ordered by the mean absolute SHAP values and their distribution.

The SHAP summary plots of top 20 features with the greatest contribution to classification in the multilevel local and network measure models are presented in Figures 6, 7, respectively. The DC value of left postcentral had the greatest contribution to the model prediction among the local measures. ReHo values in left superior frontal cortex and VMHC in right superior parietal cortex were also important for model prediction. Among the network measures, FC between the right motor ventral and left superior temporal gyrus served as the most important feature. The FC between right ECN and right caudate, and FC between right salience network and right middle temporal gyrus, as well as FC between the DMN and left medial superior frontal cortex were found as important features in model prediction.

## Discussion

There is an important need to differentiate chronic mTBI and HCs, and yet, there is a lack of objective imaging biomarkers. The scientific motivation of this study was to investigate classification performance of distinction chronic mTBI patients and HCs utilizing multivariate rs-fMRI metrics. We employed voxel-wise two-sample *t*-test comparing between the groups to generate two types of resting-state features including local and network measures. To our knowledge, no previous studies have combined voxel-wise rs-fMRI metrics to distinguish mTBI patients at the level of an individual. This is well-known that the vast majority of neurological and psychiatric diseases are associated with a combination of regional and network-level brain function alteration (Fornito and Bullmore, 2015; Worbe, 2015). Furthermore, different neuroimaging modalities capture different aspects of neuropathology and hence, may provide complementary information for detecting mTBI at individual level.

In recent years, a few studies have attempted to develop a manner for making an mTBI diagnosis using brain functional imaging with ML methods. Vergara et al. (2018) used dynamic functional network connectivity (dFNC) for mTBI detection using a linear SVM algorithm. They found the highest classification performance of 92% for one of the dFNC states (Vergara et al., 2018). Also, Vivaldi et al. (2021) used electroencephalography (EEG) for two-class classification of patients with mTBI and normal subjects employing SVM and K-nearest neighbors (KNN) models and achieved the accuracy of 0.94 *via* 10-fold CV during classification. In a recent study, researchers conducted linear SVM ML algorithm using diffusion tensor imaging (DTI) including fractional anisotropy (FA) and the ratio of axial diffusivity (AD) to radial diffusivity (RD) to different patients with mTBI from HCs. They found FA of the anterior/superior corona radiata and AD/RD of the corpus callosum and internal capsule are the best features in classification with the maximum accuracy of 89% (Harrington et al., 2022). To our knowledge, this study is the first to explore multilevel rs-fMRI metrics that includes both local and network measures, in the classification of patients with chronic mTBI and HCs using a ML method. We categorized the rs-fMRI metrics to two types of local and network measures and investigated the classification performance for separate and combined metric(s) models. As for the separate metric model, almost all showed similar and moderate classification performance. However, the combined models outperformed the separate models.

Using multimodality MRI, several studies constructed SVM classifier for identification of neurological and psychiatric diseases. Vergara et al. (2017) compared the classification performance of SVM classifier using both resting-state functional network connectivity (rsFNC) and diffusion MRI in detection of mTBI patients; however, the feature selection method they used was relatively loose and they failed to include and combine several MRI modalities. Similar to our study, Pang et al. (2021) included multivariate rs-fMRI metrics including ALFF, DC, ReHo, VMHC, and FC and examined the classification performance of single and combined metric(s) models to distinguish between motor subtype of Parkinson’s disease. Identical to our results, they found improved classification performance using combined metrics model (Pang et al., 2021). Also, another study employed combination of several rs-fMRI metrics and SVM classifying patients with bipolar disorder and reported the AUC of 0.919. However, they failed to build a single model for each metric (Wang et al., 2020). In line with this, Lei et al. (2020) incorporated multimodal MRI imaging including structural and rs-fMRI data in classification study of patients with schizophrenia using SVM algorithm. They found that the combination of images and metrics enhances classification performance resulting in highest accuracy of 90.83% (Lei et al., 2020).

The most recent study utilized different rs-fMRI metrics and structural MRI including hippocampal subfield and amygdala volumes for diagnosis of patients with Alzheimer’s disease (AD). They conducted SVM and random forest (RF) ML algorithm for classification task and found that combination of the structural and rs-fMRI metrics could significantly enhance the accuracy of classification in diagnosing AD. Moreover, it was suggested that SVM classifier performs better than RF in binary classification (Khatri and Kwon, 2022). Our experiment extended the results of the previous studies showing that classification performance can be improved by combining multilevel characteristic of rs-fMRI. Our finding demonstrated the classification accuracy of 100% for both the combined local and network measures that can be generalized to new dataset and at the patient individual level.

In order to address the black box nature of the ML method, the SHAP methodology was employed to enable interpretation of the ML models and yield the feature importance values. It assigns numerical values indicating the magnitude and direction of feature contributions to the model prediction. The best-discriminative regions were widespread and not restricted to particular brain regions and networks across the whole rs-fMRI metrics.

Among local measures, the DC value of the postcentral gyrus served as the most important feature. The postcentral gyrus is the main part of the somatosensory cortex and higher DC values in this area represents the higher-order integration of different inputs like sensory and motor (Wang et al., 2021). ReHo in the frontal cortex was also shown as important feature in discrimination of individual patients with mTBI. Several studies have demonstrated the vulnerability of the frontal lobe in mTBI. Specifically, the medial superior frontal lobe as the main part of the DMN plays a role in cognitive processing. Cognitive impairment is often associated with patients with mTBI. Therefore, the functional activity of brain regions within the DMN plays a key role in differentiating mTBI from normal subjects. In a recent study on subjects with acute sport-related concussion, Meier et al. (2020) showed increased connectivity localized in the frontal lobe regions that are typically associated with the DMN.

Additionally, it has been shown that abnormal structure, function, and FC in frontal regions are common in individuals suffering from pain disorders including headache, migraine, and mTBI. Indeed, FC alteration in the frontal lobe is linked with pain processing including the affective and cognitive processing of pain (Schwedt et al., 2017; Lu et al., 2020b). Further, researchers have repeatedly reported that the pattern of functional disruption of the DMN is associated with psychological and emotional distress in neuropsychological diseases including mTBI, suggesting the role of the DMN as a predictive biomarker of prognosis of individual patients with mTBI (Zhan et al., 2015; Guo et al., 2019; Shi et al., 2021; Vedaei et al., 2021). In the present study, we found the superior parietal gyrus as one of the regions with a great contribution to the classification. Our results are in line with previous studies reporting abnormality of resting-state FC in fronto-parietal network in patients with chronic mTBI (Marquez de la Plata et al., 2011; Shumskaya et al., 2012; Stevens et al., 2012). The fronto-parietal network has been shown to be involved in top-down attention control that is activated when attention is shifted from self-awareness to the external environment. Several reports have described abnormality in fronto-parietal connectivity during working memory task in mTBI patients (Kasahara et al., 2011). As such, increased rs-fMRI measures might be associated with excessive cognitive fatigue frequently reported in mTBI patients (Spikman and Van Der Naalt, 2010). Another important feature for the model prediction was the Rolandic operculum. This is a region that covers parts of the frontal, temporal and parietal cortex, and abnormal FC in this area has been shown to be linked with worse psychological conditions such as anxiety and depression (Sutoko et al., 2020).

Among the network measures analysis, FC value between the motor ventral area and superior temporal gyrus has been served as the most important feature with the greatest contribution to the prediction model. The motor network and temporal gyrus have been shown as two main targets of TBI. A number of studies have shown that the frontal and temporal lobes are at increased risk of contusion in moderate to severe brain injury (Zhou et al., 2014; Vedaei et al., 2021). A recent study demonstrated increased functional and structural connectivity between subnetwork nodes in the DMN including temporal superior temporal gyrus in patients with chronic mTBI 1 year after injury (Dall’Acqua et al., 2017). Shi et al. (2021) in a study on patients with mTBI showed an alteration of rs-fMRI measures in the cerebellum and temporal lobe regions and suggested that the FC abnormality is consistent with sensory perception, movement control, and micro-motor coordination in patients after injury. Also, it has been shown that motor network functional activation and connectivity in mTBI patients with post-traumatic headache is related to patients’ response to avoid movement during pain since physical movement may worsen the pain (Lu et al., 2020b).

Functional connectivity between the ECN and right caudate also were found to be important. The DLPFC as the center of the ECN has been shown to be functionally engaged during working memory tasks (Slobounov et al., 2010; Gillis and Hampstead, 2015; Mayer et al., 2015). The caudate is a part of limbic system and basal ganglia and is defined to be engaged in emotional processing. As such, we speculate that increased FC between the DLPFC and caudate is linked with compensatory mechanism of top-down cognitive control and mood-regulating in the patient group. This finding is consistent with the result of a recent study demonstrating that rs-fMRI connectivity between DLPF and cingulate-pallidostriatal- thalamic-amygdala is correlated with depressive scores in TBI cohort (Luo et al., 2021). We also found the FC between the salience network and middle temporal gyrus as one the important features. Structural and functional abnormality of the anterior insula, the center of salience network, have been reported during acute stage of mTBI. In line with our results, Lu et al. (2020a) has shown alteration of FC between insula and temporal gyrus in patients with mTBI compared to controls as well as significant correlation between this FC and Montreal cognitive assessment (MoCA) sub-scores including orientation and abstraction scores. They speculated that accumulation of amyloid in the temporal gyrus is associated with cognitive function dysfunction in patients suffering from traumatic injury (Lu et al., 2020a).

Another important feature in our model prediction was FC between the DMN and medial superior frontal gyrus. General speaking, FC abnormality between the DMN and other brain regions has been shown in many studies in patients with neurological and psychiatric disorders including mTBI. Our findings are consistent with prior literature showing hyperconnectivity between DMN regions including posterior cingulate cortex (PCC) and regions in frontal gyrus, representing brain neuroplasticity operative in neural repair and recovery after injury. Therefore, we speculate that this hyper-connectivity is linked with compensatory reallocation and recruitment of cognitive resources (Zhou et al., 2012; Sours et al., 2013; Shumskaya et al., 2017; Li F. et al., 2020). FC between visual network and middle frontal gyrus was also served as one of the important features. The occipital gyrus in the center of visual cortex has been shown to be vulnerable to brain injury. Our findings are in line with previous literature revealing that the FC abnormality in this network is linked with visual disturbances, memory, and motor perception disturbances (Palacios et al., 2017; Shi et al., 2021). Additionally, it has been shown that mTBI symptoms are strongly correlated with between-network connectivity particularly between motor, DMN, and visual networks (Madhavan et al., 2019). Palacios et al. (2013) in a study on patients suffering from chronic mTBI showed that loss of structural connectivity in these patients is compensated for by an increased in the functional connectivity of local circuits.

Prior work in mTBI using rs-fMRI has revealed abnormal FC in several brain networks including the DMN, DMN-basal ganglia, attention-sensorimotor, fronto-parietal, and visual network. For instance, Stevens et al. (2012) in an rs-fMRI study on mTBI population used independent component analysis (ICA) comparing between the patients and normal subjects and showed abnormal FC in several brain networks including visual, motor, limbic, and several other circuits involved in executive function. They proposed that abnormalities were not included FC deficits, but also reflecting compensatory neural processes.

Our findings are in line with prior studies reporting that alterations in FC in mTBI patients are more between-network than within-network indicating disruption of communication between brain network modules in this patient population. Taken collectively, these findings confirm that the hub organization and networks might interfere with multiple integrative roles such as executive function, social cognition, internal focus of attention, divided attention, vision, memory, and language (Vakhtin et al., 2013; Madhavan et al., 2019; Meier et al., 2020; Li et al., 2022).

This study has several limitations. Considering the relatively small sample size and imbalanced proportion of the classes, future studies need to validate our findings with a larger cohort and multiple clinical centers. Also, inhomogeneity of the patient population may affect the outcomes. Future studies are needed to be more considerate involving homogenous datasets. In addition, including multimodal data such as structural, diffusion weighted, and cerebral perfusion data might provide more information assisting each other in interpretation of the model predictions following individual patient classification task. Finally, different classification ML algorithms other than SVM, and feature selection methods could be executed and compared in order to introduce the optimized classification model which ultimately can be employed in clinical practice and patient classification and diagnostic at individual level.

## Conclusion

In sum, the present study provided a comprehensive approach employing SVM in classification of chronic mTBI patients using local and network rs-fMRI measures. We showed relatively high classification performance using separate models. Specifically, DC among the whole measures showed the highest classification accuracy suggesting using this metric in classification tasks when just one measure needed to be employed. Additionally, we proposed that combined multilevel rs-fMRI metrics may improve classification accuracy relative to single models. Given the pressing need of automatic clinical tools for detecting mTBI in patients with high levels of accuracy, combining neuroimaging measures within a multivariate supervised ML framework may provide a new avenue for the diagnosis of individual patients in the clinical setting.

## Data availability statement

The raw data supporting the conclusions of this article will be made available by the authors, without undue reservation.

## Ethics statement

The studies involving human participants were reviewed and approved by the Thomas Jefferson University IRB. The patients/participants provided their written informed consent to participate in this study.

## Author contributions

FV: conceptualization, formal analysis, methodology, writing – original draft, and writing – review and editing. NM and GZ: formal analysis, methodology, and writing – review and editing. DM: conceptualization, resources, and funding acquisition. EN: formal analysis, data collection, and writing – review and editing. CH: data collection, methodology, and writing – review and editing. NW: formal analysis, supervision, and writing – review and editing. AN: conceptualization, investigation, resources, supervision, validation, and writing – review and editing. FM: conceptualization, investigation, methodology, supervision, validation, and writing – review and editing. All authors contributed to the article and approved the submitted version.

## Footnotes

1
http://rfmri.org/DPARSF

2
https://www.fil.ion.ucl.ac.uk/spm/

3
http://rfmri.org/dpabi

4
www.anaconda.com

5
scikit-learn.org
